# Dominating Biological Networks

**DOI:** 10.1371/journal.pone.0023016

**Published:** 2011-08-26

**Authors:** Tijana Milenković, Vesna Memišević, Anthony Bonato, Nataša Pržulj

**Affiliations:** 1 Department of Computer Science and Engineering, University of Notre Dame, Notre Dame, Indiana, United States of America; 2 Department of Computer Science, University of California Irvine, Irvine, California, United States of America; 3 Department of Mathematics, Ryerson University, Toronto, Ontario, Canada; 4 Department of Computing, Imperial College London, London, United Kingdom; King's College London, United Kingdom

## Abstract

Proteins are essential macromolecules of life that carry out most cellular processes. Since proteins aggregate to perform function, and since protein-protein interaction (PPI) networks model these aggregations, one would expect to uncover new biology from PPI network topology. Hence, using PPI networks to predict protein function and role of protein pathways in disease has received attention. A debate remains open about whether network properties of “biologically central (BC)” genes (i.e., their protein products), such as those involved in aging, cancer, infectious diseases, or signaling and drug-targeted pathways, exhibit some topological centrality compared to the rest of the proteins in the human PPI network.

To help resolve this debate, we design new network-based approaches and apply them to get new insight into biological function and disease. We hypothesize that BC genes have a topologically central (TC) role in the human PPI network. We propose two different concepts of topological centrality. We design a new *centrality measure* to capture complex wirings of proteins in the network that identifies as TC those proteins that reside in dense *extended* network neighborhoods. Also, we use the notion of *domination* and find dominating sets (DSs) in the PPI network, i.e., sets of proteins such that every protein is either in the DS or is a neighbor of the DS. Clearly, a DS has a TC role, as it enables efficient communication between different network parts.

We find statistically significant enrichment in BC genes of TC nodes and outperform the existing methods indicating that genes involved in key biological processes occupy topologically complex and dense regions of the network and correspond to its “spine” that connects all other network parts and can thus pass cellular signals efficiently throughout the network. To our knowledge, this is the first study that explores domination in the context of PPI networks.

## Introduction

A *network* (or a *graph*) is a set of *nodes* (or *vertices*), and *edges* (or *links*) between the nodes. Networks enable studying the properties of complex systems that emerge from interactions among individual parts. Hence, networks have been used to model and analyze many real-world phenomena in numerous domains. Examples include social, technological, transportation, information, financial, ecological, chemical, and biological systems. We focus on molecular interaction networks, with the goal of understanding complex cellular functioning by studying cells as inter-connected systems rather than as a collection of individual constituents [1]. Nodes in these networks represent biomolecules, such as genes, proteins, or metabolites, and edges connecting the nodes indicate functional, physical, or chemical interactions between the corresponding biomolecules. Since proteins execute the genetic code and carry out most biological processes, we focus on protein-protein interaction (PPI) networks. In these networks, nodes correspond to proteins and undirected edges represent physical interactions between them.

We have been witnessing the exponential growth of the amounts of available PPI network data, along with the development of computational approaches for studying and modeling of these data. High-throughput screens for interaction detection, such as yeast two-hybrid (Y2H) assays [2]–[8], affinity purification coupled to mass spectrometry (AP/MS) [9]–[12], genome-wide chromatin immunoprecipitation, correlated m-RNA expression, and genetic (synthetic-lethal) and suppressor networks [13], [14], have yielded partial networks for many model organisms [2]–[5], [11]–[13] and humans [6], [7], as well as for bacterial [15]–[17] and viral [18]–[20] pathogens. Numerous biological network datasets are now publicly available in several databases, including Saccharomyces Genome Database (SGD) [21], the Database of Interacting Proteins (DIP) [22], Human Protein Reference Database (HPRD) [23], and the Biological General Repository for Interaction Datasets (BioGRID) [24].

Proteins are essential macromolecules of life, and hence, understanding their function and their role in disease is of importance. Since proteins aggregate to perform a function instead of acting in isolation, and since PPI networks model interactions between proteins, analyzing PPI network topology is expected to uncover new biology. Therefore, it is not surprising that prediction of protein function [25]–[27] and the role of protein networks in disease [1], [28]–[32] from the topology of PPI networks have received attention in the post-genomic era.

Nonetheless, there is still a debate about whether network properties of “biologically central” genes or proteins, such as those involved in aging, cancer and infectious diseases caused by bacterial or viral pathogens (*e.g.*, HIV, herpesvirus, hepatitis, and influenza), exhibit some “topological centrality” compared to the rest of the proteins in the PPI network [1], [28]–[31], [33]–[35]. Many approaches have focused on examining only simple topological properties of these proteins, such as their direct neighborhoods in a PPI network. For example, the key assumption of many studies is that proteins that are direct neighbors are more likely to perform the same function than those that are not [25], [26], or that a neighbor of a disease-causing gene is likely to cause either the same or a similar disease [1], [34]. Another example is the observed correlation between a protein's essentiality and its *degree centrality* (the larger the degree of a node, the more “degree-central” the node) in a PPI network of baker's yeast [36]. However, the controversy arose in the light of newer and more complete PPI network data for which this correlation was not observed [37], [38] and it appears to hold only for literature-curated [39] and smaller in scope Y2H PPI networks [3], possibly because these data sets are biased towards essential proteins [38]. Also, degree alone might be a weak measure of network topology, as it captures limited network topology, i.e., only direct neighborhood of a node [27], [31], [40]. A similar controversy arose when cancer genes were initially shown to have greater connectivities and centralities compared to non-cancer genes, indicating central roles of cancer genes within the interactome [33], but it was later demonstrated that most of disease genes do not show a tendency to code for proteins that are hubs [29], although a recent study again reached the conclusion that cancer proteins have different network topologies, e.g., higher degrees, than “control” genes [35]. Apart from this, general conclusions are that disease genes have high connectivity and are centrally positioned within the PPI network [1]. In addition, it has been suggested that aging genes tend to have higher degrees than non-aging ones [41], [42], as well as that the majority of viral and bacterial pathogens show tendency to interact with high-degree proteins, or with “bottleneck” proteins that are central to many paths in the PPI network [43].

Measures of network topology that are more constraining than degrees might help resolve these controversies. Hence, various topological centrality concepts have been formulated. Examples include the *betweenness centrality* [35], according to which nodes that occur in many of the shortest paths in a network have high centrality, and the *subgraph centrality*, which counts the number of closed walks of different lengths in the network starting and ending at the node in question and according to which nodes that participate in a large number of such walks have high centrality [44], [45].

In addition, we have recently designed a graphlet-based measure of network topology; graphlets are small *induced* subgraphs of a large network [46], [47]. As opposed to *partial* subgraphs (e.g., network *motifs* [48]), graphlets are *induced*, meaning that they contain *all* edges between the nodes of the subgraph that are present in the large network. This measure generalizes the degree of a node that counts the number of edges that the node touches, where an edge is the only 2-node subgraph, into the *graphlet degree vector* (GDV) that counts the number of different graphlets that the node touches, for all 2–5-node graphlets. Hence, GDV of a node describes the topology of its up to 4-deep neighborhood. This is an effective measure: going to distance of 4 around a node captures a large portion of a network due to the small-world nature of many real networks [49]. For this reason, and since the number of graphlets on 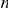 nodes increases exponentially with 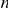, we believe that using larger graphlets would unnecessarily increase the computational complexity of the method. We designed the similarity measure between GDVs of different nodes, *GDV-similarity*, to quantify the topological similarity of the extended neighborhoods of two nodes. We used this constraining measure of network topological similarity to demonstrate that: in PPI networks, biological function of a protein and its local network structure are closely related [27], [50]; from topology of PPI networks we can extract biological information that cannot always be extracted from sequence and hence, topology could be used as a complementary method to sequence-based methods for homology detection [51]; topology around cancer and non-cancer genes is different and can be used to successfully predict new cancer genes in melanogenesis-related pathways [31], [40]; purely topological network alignments can be used to extract protein function and species phylogeny [52], [53].

### This study

Here, we present novel network-based approaches applied towards a deeper understanding of biological function and disease. We aim to further study and understand currently poorly described mechanisms by which “biologically central” genes interact with each other and with other genes in the cell. We define as *biologically central (BC)* the genes that belong to one of the following four gene *categories*: aging (A) genes, cancer (C) genes, HIV-interacting (HIV) genes, and pathogen-interacting (PI) genes. Our hypothesis is that BC genes, i.e., their protein products (henceforth, we use terms “gene” and “protein” interchangeably), will have a topologically central role in the human PPI network. We use two different concepts to define “topological centrality”: *graphlet degree centrality* and *domination* (defined below).

**Figure 1:**
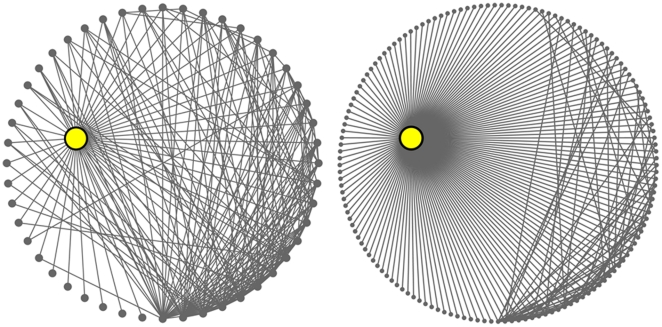
An illustration of the differences between DC and GDC. *Left:* Direct neighborhood of ZAP90, a cancer and HIV gene, in the human PPI network [28]. Its degree is 48 and it is ranked as the top 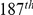 gene with respect to DC. *Right:* Direct neighborhood of PRKACA, an HIV gene, in the network. Its degree is 145 and it is ranked as the top 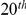 gene with respect to DC. Both proteins have the same GDC and are ranked as top 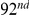 genes with respect to GDC. Hence, GDC rewards the ranking of a low-degree gene if its 4-deep neighborhood is dense (ZAP90) and penalizes the ranking of a high-degree gene if its 4-deep neighborhood is sparse (PRKACA). (For the esthetics of the figure, we only show 1-deep neighborhoods.)

Previously, we defined GDV-similarity of nodes' neighborhoods that is independent of the densities of these neighborhoods: nodes with identical GDVs have the maximum GDV-similarity, regardless of whether they reside in dense or sparse neighborhoods. Here, we propose a new centrality measure, *graphlet degree centrality (GDC)*, to measure the density and complexity of nodes' neighborhoods by counting the number of different graphlets that the node touches. According to GDC, nodes in dense and complex 4-deep neighborhoods will have higher centralities than nodes in sparse 4-deep neighborhoods. GDC is a different and more constraining measure of network topology than the degree centrality (DC), as illustrated in Figure 1: GDC ranks highly a low-degree gene if its 4-deep neighborhood is dense and gives a low rank to a high-degree gene if its 4-deep neighborhood is sparse (details are below). GDC is conceptually different than the betweenness centrality (BWC), which does not measure topological denseness at all. Subgraph centrality (SC) measures the number of closed walks (which can be thought of as partial subgraphs) that the node touches and it has been shown to be more highly correlated with the lethality of proteins in the PPI network of baker's yeast than DC [44]. Unlike SC, GDC counts induced subgraphs rather than partial ones and in a more rigorous way: while SC counts an edge that a node touches many times, as a 2-edge closed walk (going from node A to node B along edge AB and returning from B to A along the same edge), as a 4-edge closed walk (going from node A to node B and back to A twice), as a 6-edge closed walk (going from A to B and back to A three times) etc., GDC counts the edge only once and only as an edge, rather than as different subgraph structures.

For each of the four centrality measures (DC, BWC, SC, and GDC), we identify the most central genes (explained below) in the human PPI network [28] and measure the *enrichment* of these genes in BC genes (i.e., the percentage of the most central genes that are BC genes), with the goal of finding the centrality measure that is the most discriminative in uncovering BC genes; ideally, the most discriminative measure would have all of the most central genes to be BC genes. We find that: (1) enrichments in BC genes of the most GDC-central genes are much higher than those of non-GDC-central genes, (2) the observed enrichments in BC genes of the most GDC-central genes are statistically significant, while those of non-GDC-central genes are not, (3) BC genes that are GDC-central have higher and statistically significant enrichments in known drug targets than BC genes that are non-GDC-central, and (4) GDC is at least as discriminative as the next best centrality measure.

Second, we hypothesize that genes that are vital for normal cellular functioning might correspond to the “spine” of the network that connects all parts of the network. The field of telecommunications and the domain of the efficient design of routing protocols for wireless networks in particular, uses the notion of a *dominating set* (DS) to find the most central set of nodes in wireless networks that would be used for efficient data routing and lead to bandwidth increase and energy savings; in wireless networks, nodes correspond to computers and routers, and edges correspond to links between them [54]–[57]. A dominating set of a network is a set of nodes such that every node in the network is either in the DS or is a direct neighbor of a node in the DS. Hence, the nodes in the dominating set act as a “gateway” in the network, since all nodes in the network are at most one step away from them and the transfer of the information to all nodes can be quick and cheap. The challenge is to identify a minimum order DS, a DS of the minimum size (i.e., the minimum number of nodes). This problem is NP-hard. Thus, approximate (heuristic) algorithms are sought.

Given the topologically central role of nodes in a DS, we hypothesize that a good DS algorithm might capture a set of proteins in a PPI network that are involved in important biological processes and mechanisms crucial for cell vitality, i.e., that DSs of PPI networks might contain BC proteins and signaling pathways (SPs). We test this by constructing a connected dominating set in the human PPI network with an algorithm that is commonly used in telecommunications [57]. We are interested in connected DSs only since signaling pathways are connected. Other algorithms for finding connected DSs are used in telecommunications as well (e.g., [54], [56], [58], [59]), but are not applicable to biological networks, because they require nodes to be assigned meaningful numerical IDs, e.g., IP addresses in computer networks; clearly, proteins in PPI networks do not have numerically meaningful labels. Also, several algorithms for finding disconnected (i.e., independent; see Methods) DSs exist [60], [61], but they are inappropriate for our study for the above mentioned reasons. In addition to applying the existing DS algorithm of Rai *et al.* [57], we design a new and simpler DS algorithm that outperforms the algorithm of Rai *et al.* on our data (explained below). Note that the main focus of this study is not to create a state-of-the-art algorithm for finding DSs, but instead, to demonstrate, as a proof of concept, that a DS of a PPI network found by a very simple algorithm indeed captures biologically vital proteins. Any further algorithmic improvements are likely to yield more optimal DSs and hence improve the biological results.

We apply DS algorithms to the human PPI network [28] and measure the size of the resulting DSs, as well as their enrichments in BC and SP genes. We find that: (1) the enrichments in BC and SP genes of nodes of DSs are much higher than the enrichments of nodes outside of DSs; (2) the enrichments in BC and SP genes of nodes of DSs are statistically significant, while those of nodes outside of DSs are not; and (3) BC and SP genes that are in DSs have much higher and statistically significant enrichments in known drug targets than BC and SP genes that are not in DSs. Hence, we confirm our hypothesis that DSs capture biologically vital proteins and also drug targets.

Furthermore, we demonstrate not only that each of the two measures of topological centrality, GDC and DS, captures a statistically significant biological signal, i.e., BC and drug target genes (as described above), but also that the combination of the two centralities is even more discriminative in capturing these genes. To our knowledge, this is the first study that uses dominating sets to analyze PPI networks.

## Methods

### Data sets

We analyze the human PPI network of Radivojac *et al.* that contains 41,456 physical interactions between 9,141 proteins [28], as well as the human PPI networks from BioGRID [24], that contains 30,513 physical interactions between 8,581 proteins, and from HPRD [23], that contains 36,811 physical interactions between 9,449 proteins (we downloaded them in June 2010). Since we obtained qualitatively similar results for all three networks, for simplicity we report only on the PPI network of Radivojac *et al.* [28]; we chose this network, since it has the largest number of interactions.

As mentioned above, *biologically central (BC)* genes that we analyze include: aging, cancer, HIV, and pathogen-interacting genes. We obtained them from the following databases. *Aging genes (A)* are human genes implicated in the process of aging that are available from AnAge Databank - Human Ageing Genomic Resources (http://genomics.senescence.info/) [62]. *Cancer genes (C)* are human genes implicated in cancer that are available from: Cancer Gene Database (http://ncicb.nci.nih.gov/projects/cgdcp), Cancer Genome Project – the Cancer Gene Census (http://www.sanger.ac.uk/genetics/CGP/Census/) [63], GeneCards (http://www.genecards.org/) [64], Kyoto Encyclopedia of Genes and Genomes (KEGG) (http://www.genome.jp/kegg/disease/) [65], and Online Mendelian Inheritance in Man (OMIM) (http://www.ncbi.nlm.nih.gov/sites/entrez?db=omim) [66]. *HIV genes (HIV)* are human genes known to interact with genes of the HIV virus [63] that are available from HIV-1-Human Protein Interaction Database (http://www.ncbi.nlm.nih.gov/RefSeq/HIVInteractions/) [67]. Finally, *pathogen-interacting genes (PI)* are human genes known to interact with genes of pathogens [43]. The data are downloaded in 2009 and 2010.

**Figure 2:**
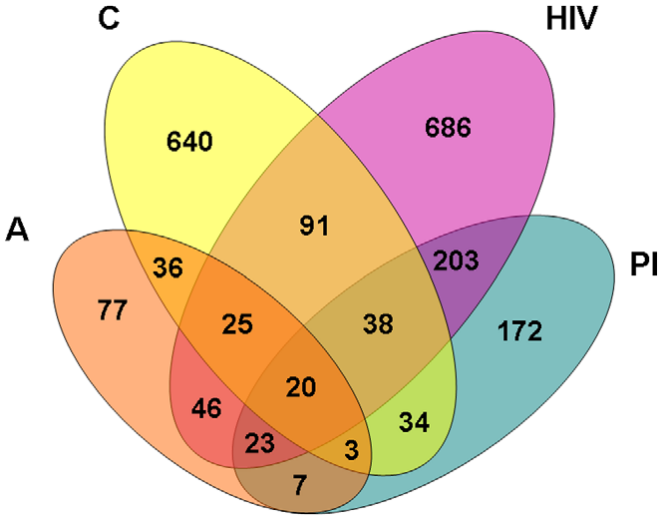
The overlap of BC genes from the four categories in the human PPI network.

In the human PPI network, there are 2,101 BC genes in total, of which 237 are aging genes, 887 are cancer genes, 1,132 are HIV genes, and 500 are PI genes. Figure 2 illustrates the overlap of different BC gene categories in the network. The overlap is low and there are only 20 BC genes that are simultaneously aging, cancer, HIV, and PI genes.

Signaling pathways (SPs) that we analyze include the human: MAP kinase interactome [68], cancer and immune pathways from NetPath [69], and all human signaling pathways from KEGG [65]. The data are downloaded in November 2010. In the PPI network, there are 2,253 SP genes, 911 of which are also BC genes. Given that there is a total of 2,101 BC genes in the network, the total number of BC and SP genes together is 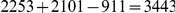.

The drug target data was downloaded from DrugBank [70].

### Centrality measures

#### Related work

Several notions of node centrality have been used in the past. *Degree centrality (DC)* of a node is the number of its neighbors, i.e., its degree. Alternatively, DC can be normalized by dividing the degree with 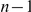, where 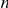 is the number of nodes in the network. *Betweenness centrality (BWC)* of a node is the sum, over all node pairs 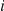 and 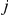 in the network, of the percentage of all shortest paths between 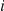 and 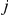 in the network that go through the node of interest. *Subgraph centrality (SC)* of a node is a weighted sum of the numbers of all closed walks of different lengths in the network starting and ending at the node. These closed walks are related to partial subgraphs of a network, e.g., a closed walk with four nodes can “go through” different subgraphs on four nodes, such as along the same edge AB twice (as described above: from node A to node B along edge AB, then back to A along the same edge and then again from A to B and back to A along the same edge), or along a 4-node cycle ABCD that includes edge AB (along the “square” from node A to node B to node C to node D and back to A; this is regardless of whether edges CA and DB that “go along the diagonal of the square” exist) etc. The above mentioned sum is weighted so that the contribution of the closed walks decreases as the length of the walks increases, i.e., shorter walks (smaller subgraphs) have higher weight.

#### Graphlet degree centrality

**Figure 3:**
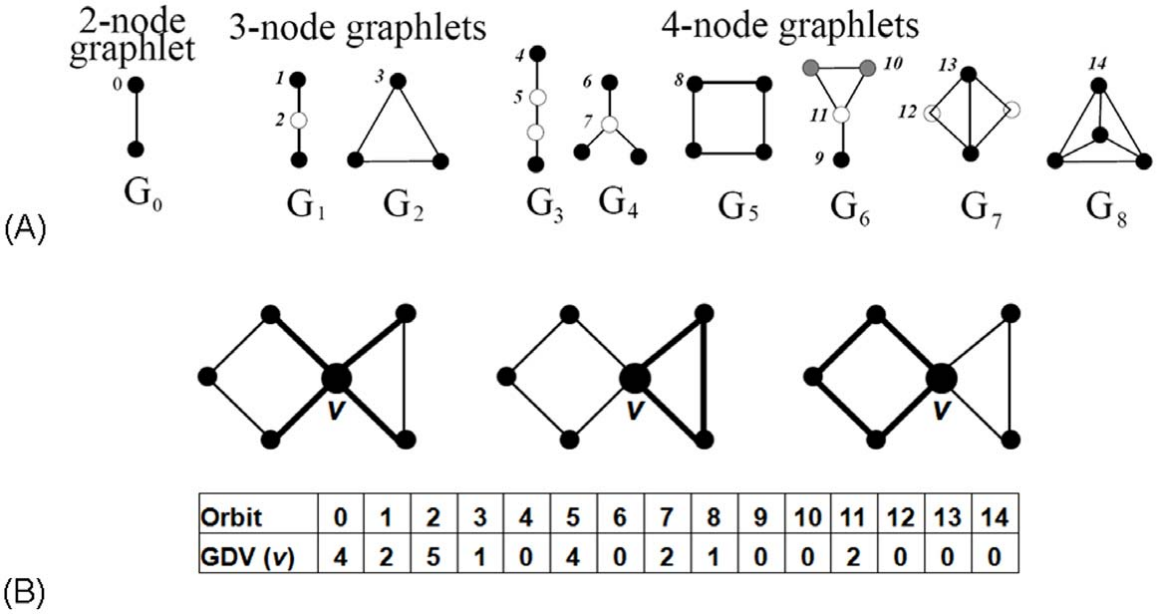
Graphlets, automorphism orbits, and GDVs. (**A**) All 9 graphlets with 2, 3 and 4 nodes, denoted by 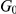, 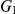,…,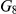; they contain 15 topologically unique node types, called automorphism orbits, denoted by 0, 1, 2, …, 14. In a particular graphlet, nodes belonging to the same orbit are of the same shade (see [47] for details). (**B**) An illustration of the GDV of node 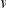; it is presented in the table for orbits 0 to 14: 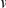 is touched by 4 edges (orbit 0), end-nodes of 2 graphlets 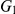 (orbit 1), etc. The figure is taken from [53].

We introduce a new node centrality measure as follows. *Graphlets* are small, connected, induced, non-isomorphic subgraphs of a large network (Figure 3 A) [46], [47]. Previously, we generalized the degree of a node, that counted how many edges the node touched, into the *graphlet degree vector (GDV)*, that counted how many graphlets of a given type, such as a triangle or a square, the node touched (Figure 3 B) [27]. In Figure 3 B, this is illustrated by a node being touched by an edge (the leftmost illustration), a triangle (the middle illustration), or a square (the rightmost illustration). More precisely, coordinates of a GDV count how many times a node is touched by a particular symmetry group (*automorphism orbit*, see [47] for details) within a graphlet (Figure 3 B). Clearly, the degree of a node is the first coordinate in GDV, since an edge is the only 2-node graphlet. There is a total of 73 orbits in all 2–5-node graphlets. Thus, the GDV of a node, describing its up to 4-deep neighborhood (i.e., 2–5-node graphlets around it), has 73 coordinates [27]. An example of a GDV of a node that contains all 73 orbits can be found in [52].

We introduce a new node centrality measure, *graphlet degree centrality (GDC)*, which measures the density of the node's extended network neighborhood. Hence, nodes that reside in dense extended network neighborhoods will have higher GDCs than nodes that reside in sparse extended network neighborhoods. In particular, we define GDC as follows. For a node 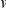, we denote by 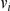 the 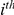 coordinate of its GDV, i.e., 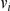 is the number of times node 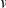 touches an orbit 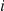. Then, GDC of node 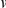 is computed as follows:
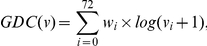
where 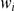 is the weight of orbit 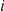 that accounts for dependencies between orbits, as in [27]; e.g., counts of orbit 3, a triangle, will affect counts of all orbits that contain a triangle. Hence, for each orbit, we count how many orbits affect it and assign a higher weight 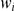 (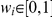) to the orbits that are not affected by many other orbits (see [27] for details). We use 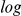 in the formula because the coordinates 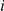 and 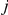 of the GDV of node 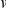 can differ by several orders of magnitude and we do not want the GDC to be entirely dominated by orbits with very large values. We add 1 to 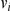 in the formula to prevent the logarithm function to go to infinity for an orbit count of 0. Finally, we scale the value of the 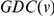 to (0,1] by dividing it with the maximum 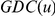 over all nodes 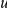 in the network.

### Algorithms for finding dominating sets

Let 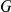
(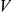,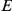) be a network, where 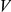 is the set of nodes of 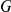 and 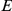 is the set of edges of 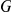. A *dominating set (DS)* of graph 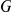 is a subset 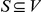 of the nodes such that for all nodes 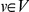, either 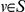 or a neighbor 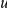 of 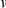 is in 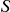. A dominating set is said to be *minimal* if it contains no proper subset that is dominating and it is said to be *minimum* if it is of the smallest cardinality. The cardinality of a *minimum* dominating set of graph 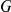, 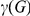, is called the *domination number* of G. It has been shown that for graph 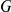 with 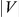 nodes:
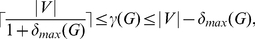
(1)where 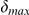 (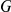) is the maximum node degree in G [60]. Identifying a minimum DS is NP-hard, and hence, approximate (heuristic) algorithms are sought.

Heuristic algorithms result in either an independent DS or a connected DS. A subset 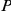 of 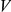 is said to be an *independent set* if no two vertices in 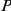 are adjacent. A connected DS is a DS in which each node is connected to at least one other node that is in the DS. (Note that if a graph consists of several connected components, a DS of such a graph would be connected within each component, but disconnected across components.) In the context of biological networks, we are interested in connected DSs.

**Figure 4:**
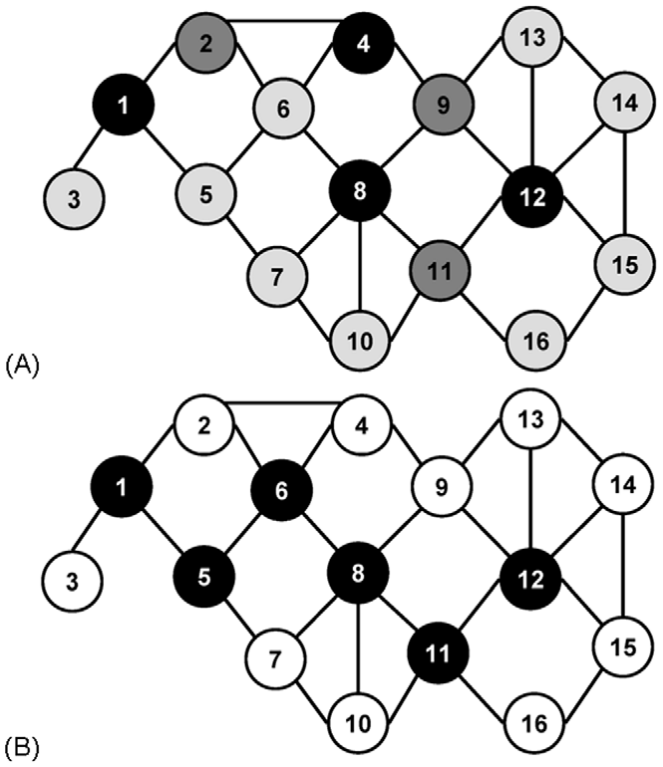
An illustration of DSs in a toy network. The DSs were obtained by (**A**) DS-RAI and (**B**) DS-DC algorithms. The example in panel A is taken from [57], and the authors describe the algorithm as follows. In phase 1, nodes 1, 4, 8, 12, and 16 are colored black as members of an independent DS. In phase 2, nodes 2, 9, and 11 are colored dark grey as connectors that connect nodes in the independent DS resulting from phase 1. In phase 3, the connected DS resulting from phase 2 is pruned to reduce it size by removing node 16 from the DS (no other nodes can be removed without violating the requirement of producing a connected DS of the graph). In panel B, all nodes are initially in the DS and then nodes are visited in order of their increasing degrees and removed from the DS if the resulting DS is a valid connected DS of the graph. That is, nodes are removed in the following order: 3, 16, 2, 4, 7, 10, 13, 14, 15, and 9. The resulting DS therefore contains the remaining nodes: 1, 5, 6, 8, 11, and 12. Clearly, the DS produced by DS-DC (black nodes in panel B) is smaller than the DS produced by DS-RAI (black and dark grey nodes in panel A).

First, we implement an existing algorithm by Rai *et al.* for constructing a connected DS of graph 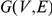 that is commonly used in telecommunications [57]. We call this algorithm “DS-RAI”. It consists of three phases: (1) constructing an independent DS named 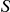, (2) finding a set of nodes 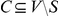 to connect nodes in 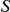 by constructing the Steiner tree between the nodes in 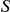, and (3) pruning the DS defined on nodes 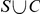 to reduce the number of nodes in the DS. More specifically, the algorithm works as follows. In phase 1, each node is colored white. A white node 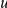 that is connected to most other white nodes is taken from 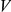, colored black meaning that it is a “dominator,” and added to 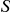. All neighboring nodes of 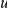 are colored gray meaning that they are “dominatees” and added to 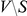. Previous steps are repeated on the remaining white nodes in 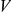 until all nodes of 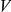 are either colored black and added to 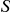, or colored gray and added to 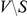. In phase 2, a gray node from 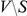 that is connected to the largest number of black nodes in 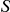 is selected, colored dark gray meaning it is a “connector,” and added to 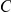. The algorithm then checks whether node set 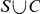 is connected and if so, it stops; otherwise, the algorithm selects the next gray node from 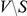 that is connected to the largest number of black nodes in 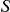 and repeats the entire process until node set 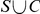 becomes connected. In phase 3, “redundant” nodes are deleted from the connected DS defined on 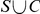 to reduce its size as follows. Let 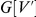 denote a subgraph of 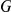 induced on a subset of nodes 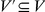. The algorithm selects a node 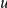 with the minimum degree in 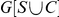 and checks whether the DS defined on 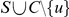 remains a connected DS of G. If so, the node 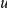 is removed from 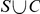. Otherwise, it remains in 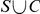. This is repeated for all nodes in 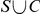, in the order of their increasing degrees. The node set resulting from node removals from 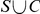 in step 3 is the final DS produced by DS-RAI algorithm. An illustration is presented in Figure 4 A.

The algorithm breaks all ties uniformly at random. Interestingly, the algorithm is robust to this randomness: we run the algorithm on the human PPI network 30 times using different random seeds, which results in 94.2% overlap between the resulting 30 DSs. The average DS size over the 30 runs is 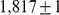 nodes, out of which 1,711 (i.e., 94.2%) appear in all of the 30 DSs. Hence, given that such a large proportion of any DS is in all DSs, any DS is representative of all of them. Therefore, we continue further analyses of one of the DSs.

Next, we introduce a new, simple, one-step algorithm for constructing a connected DS, that we call “DS-DC”: it starts with 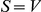, selects a node 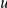 with the minimum degree in 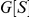, removes 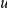 from 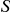 only if the DS defined on 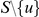 remains a connected DS of G, and repeats the above steps for all nodes in 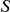 in order of their increasing degrees. An illustration is presented in Figure 4 B. Clearly, DS-DC is much simpler than DS-RAI. Also, as illustrated in Figure 4, DS-DC results in a smaller DS than DS-RAI (the same holds for real-world PPI networks, as demonstrated in Section 0). Finally, we introduce a modification of DS-DC in which nodes from 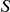 are visited in order of their increasing GDCs instead of degrees, which we call “DS-GDC” algorithm.

### Statistical significance of enrichments

For a given protein set 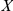 of size 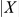, we measure its enrichment in BC (and SP) genes. We compute the statistical significance (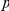-value) of observing a given enrichment by measuring the probability that the same enrichment would be observed in a randomly chosen set of 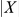 proteins in the PPI network. This probability is computed as follows by using the following notation: the total number of proteins in the network is 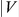; the number of proteins in set 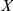 is 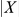; the number of proteins in set 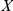 that are BC (SP) genes is 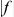; there are 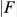 proteins in the entire PPI network that are BC (SP) genes. Then, the enrichment is 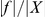, and the 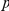-**value**, i.e., the probability of observing the same or higher enrichment purely by chance, is obtained by using the hypergeometric distribution formula for sampling without replacement:
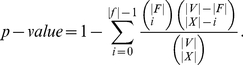
(2)


## Results and Discussion

### GDC captures BC genes

**Figure 5:**
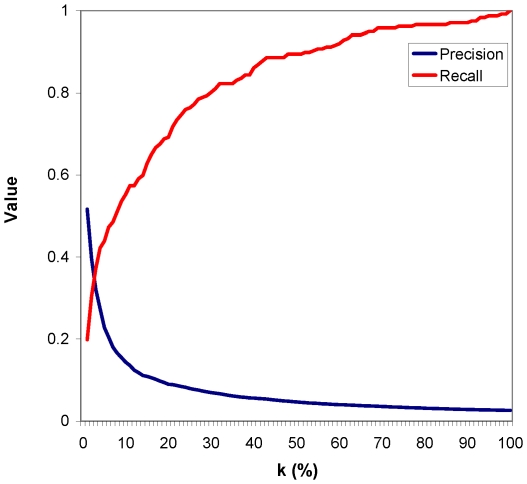
Precision and recall for aging genes in the human PPI network. They were computed for the top k% of the most GDC-central genes (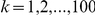). Here, precision and recall cross at 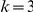.

For each of the four centralities (DC, BWC, SC, and GDC) and each of the four categories of BC genes (A, C, HIV, and PI), we find in the human PPI network the top 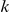
% of the most central genes (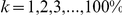) and measure how many BC genes they contain. For example, we measure how many cancer genes (C) are in the top 1%, the top 2%, the top 3% etc. most central genes with respect to each of the four centrality measures. We do the same for aging (A), HIV, and PI genes. For a given centrality measure, BC gene category, and 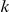, we quantify the accuracy of the centrality measure in capturing BC genes by computing precision and recall. Precision can be seen as a measure of exactness: it is the percentage of the top 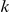
% of the most central genes that are BC genes. Recall can be seen as a measure of completeness: it is the percentage of BC genes of the network that are in the top 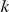
% of the most central genes. We need to determine a threshold for 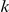 that results in the best combination of precision and recall. Since when varying the values of 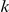, every decrease in precision corresponds to increase in recall, we choose as the threshold for 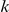 the point where precision and recall cross (Figure 5). We do this for each of the four centrality measures and each of the four BC gene categories. If the threshold is found to be 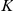, we denote as “central” those genes that are amongst the top 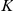
% of the most central genes and as “non-central” all the remaining genes in the network. We find that the thresholds are 3, 10, 12, and 6, for A, C, HIV, and PI genes, respectively, for each of the four centrality measures.

**Figure 6:**
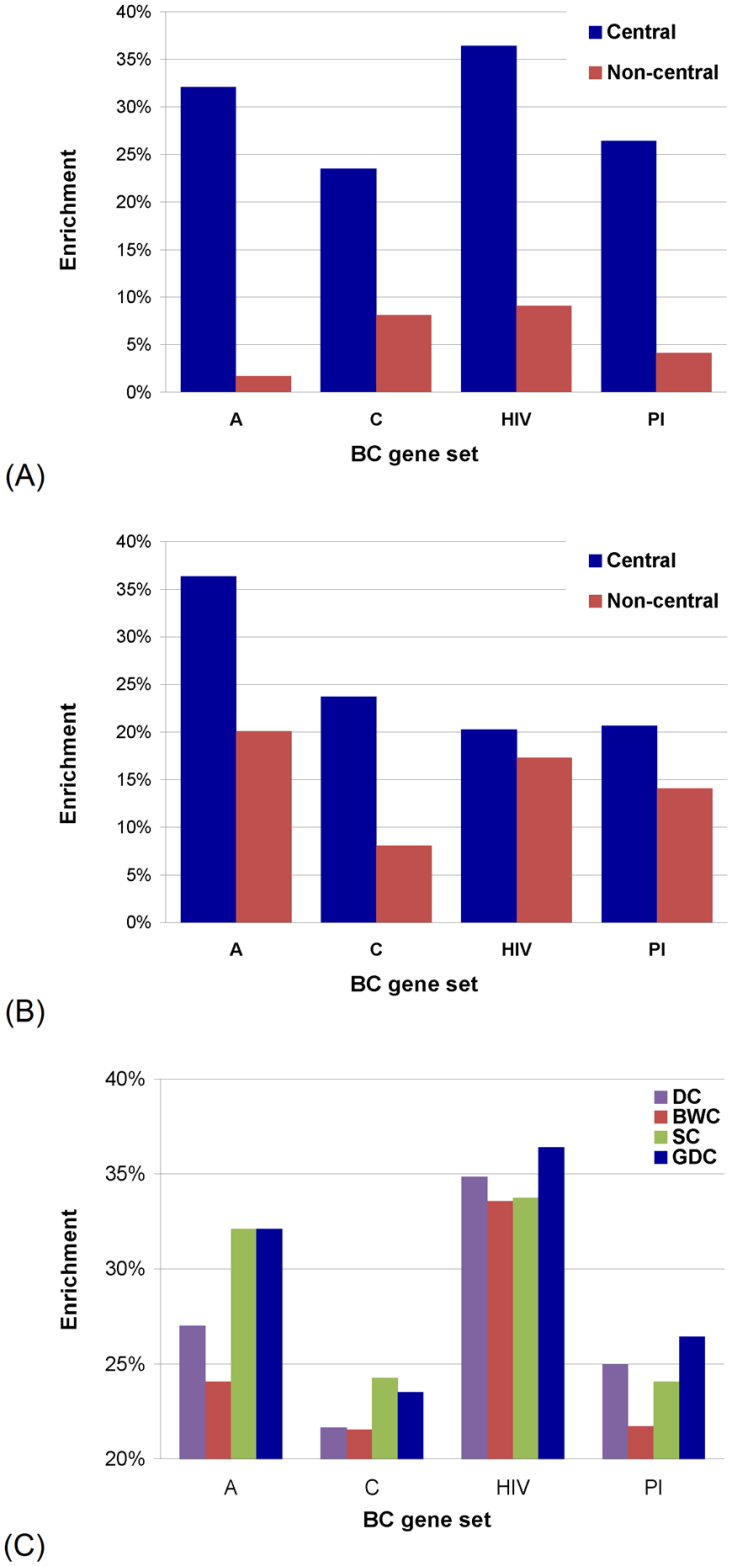
The performance of GDC and its comparison with other centrality measures. (**A**) Enrichments in BC genes of the top 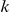
% of the most GDC-central genes (denoted by “Central”, blue bars) and all remaining genes (denoted by “Non-central”, red bars) in the human PPI network. (**B**) Enrichment in drug targets of BC genes that are GDC-central (“Central”) and BC genes that are non-GDC-central (“Non-central”). (**C**) Enrichments in BC genes of the top 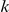
% of the most central genes in the human PPI network, with respect to the four centrality measures (DC, BWC, SC, and GDC), broken into the four BC gene categories (aging (A), cancer (C), HIV (HIV), and pathogen-interacting (PI) genes). In all panels, the values of 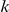 where precision and recall cross (as illustrated in Figure 5) are used; 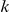 equals 3, 10, 12, and 6, for A, C, HIV, and PI genes, respectively, for each of the four centrality measures.

**Figure 7:**
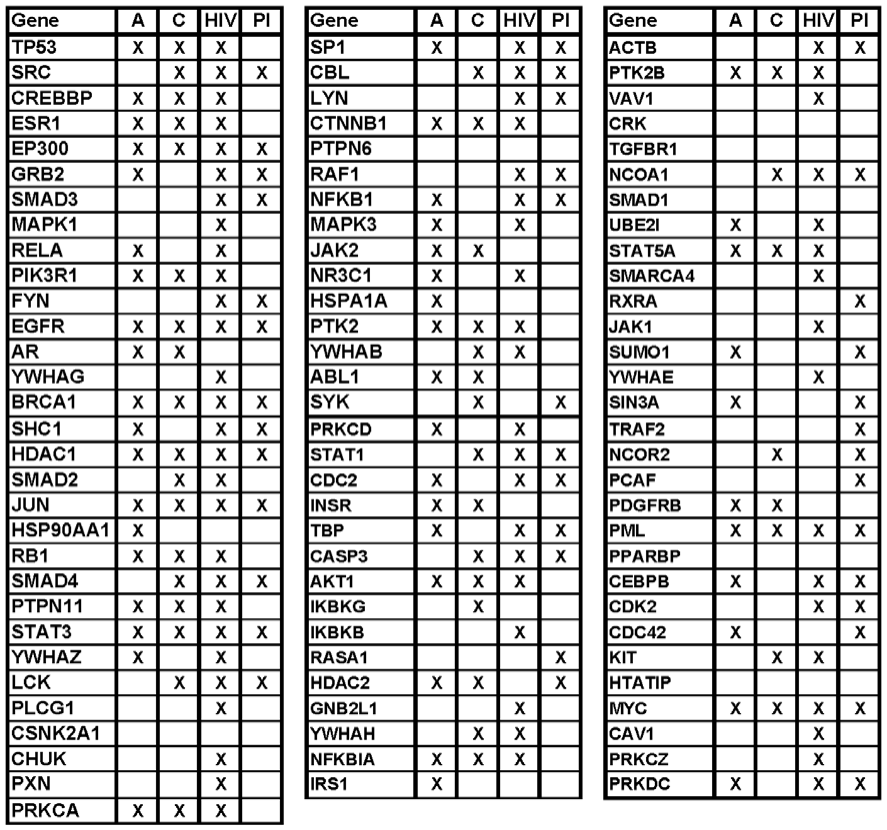
The top 1% (i.e., 91) GDC-central genes. If a gene is an aging (“A”), cancer (“C”), HIV (“HIV”), or pathogen-interacting (“PI”) gene, there is an “X” in the corresponding entry.

We compute the BC gene enrichments of central and non-central genes. We find that with respect to GDC, enrichments in each of the four BC gene categories are much higher for central genes, ranging between 23.5% and 36.4%, than enrichments for non-central genes, ranging between 1.6% and 9.5% (Figure 6 A). These enrichments are statistically significant for central genes, with 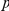-values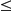 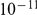, while for non-central genes they are not, with 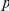-values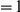 (see Methods). As expected, if we choose lower 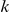, e.g., 1%, precision is even higher (although recall is lower): out of the top 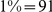 of the most GDC-central proteins in the network, 55% (i.e., 47 of them) are aging genes, 45% (i.e., 41 of them) are cancer genes, 71.5% (i.e., 65 of them) are HIV genes, and 42.9% (i.e., 39 of them) are PI genes (Figure 7).

Also, we measure the enrichment in drug targets of BC genes (i.e., of each of the four BC gene categories: “A”, “C”, “HIV”, and “PI” defined above) that are GDC-central and of BC genes that are non-GDC-central. We hypothesize that higher GDC of nodes in the PPI network reflects their functional importance. Proteins that are targeted by drugs are clearly functionally important. Hence, we examine whether the sets of BC genes that are GDC-central contain more drug targets than the sets of BC genes that are non-GDC-central. Indeed, we find that enrichments in drug targets are higher for BC genes that are GDC-central than for BC genes that are non-GDC-central (Figure 6 B). Furthermore, these enrichments in drug targets are statistically significant for GDC-central BC genes (with the exception of GDC-central HIV genes), with 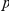-values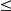 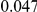, while for non-GDC-central BC genes they are not, with 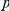-values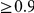 (see Methods).

In addition to the above demonstration that GDC captures statistically significant biological signal, we compare its performance against the performance of the three other centrality measures (DC, BWC, and SC). We do so by determining which measure is the most discriminative in the sense that it uncovers the largest number of BC genes amongst the top 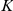
% of the most central genes (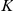 is computed as above) and hence results in the highest enrichments. As shown in Figure 6 C, GDC is at least as good as other centrality measures for all categories of BC genes, except for cancer genes, for which SC has a slightly higher enrichment, but GDC still outperforms DC and BC. GDC always outperforms DC, confirming our hypothesis that GDC, as a more constraining measure of network topology, could capture the biological signal better. SC also outperforms DC for aging genes, but interestingly not for HIV and PI genes. Hence, although GDC and SC both capture deeper network topology than DC and are conceptually similar in the sense that they both count a number of subgraphs that a node participates in, unlike GDC, SC is not always more discriminative than DC.

To evaluate whether GDC captures statistically significant biological signal and outperforms other centrality measures irrespective of the chosen thresholds 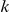, for each centrality measure, we compute the area under precision-recall curve (AUPR) as the threshold is varied between 0% and 100% in increments of 1%. The results obtained from AUPRs corresponding to different centrality measures are mostly consistent with the results obtained at selected thresholds where precision and recall cross (described above): for HIV and PI genes, AUPRs for GDC are the highest, followed by AUPRs for DC, SC, and BWC, respectively; for A and C genes, AUPRs for SC are the highest, followed by AUPRs for GDC, DC, and BWC, respectively. Hence, as was the case for individual thresholds (see above), GDC always outperforms DC, while SC outperforms DC only for A and C genes. Hence, GDC is always more discriminative than DC, while SC is not always more discriminative than DC, even though SC captures a deeper network topology compared to DC. The values of AUPRs for GDC are: 0.27 for A, 0.2 for C, 0.34 for HIV, and 0.2 for PI genes. These somewhat law values are not surprising, since in biological applications, the number of positive examples (here, the known BC genes) is much smaller than the number of negative examples (here, all proteins in the network that are currently not known to be BC genes). Furthermore, we do not know true negatives (genes that are true non-BC genes). Since we expect that many currently unreported BC genes will turn out to be BC genes in the future, AUPRs are likely to increase as this happens. Moreover, the observed AUPRs are statistically significant: we compute, at each value of recall, the probability of observing a given precision and we find that the probabilities of observing a given number of BC genes among 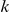
% of randomly chosen genes are in the range 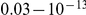 for 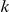 up to 90% (clearly, for 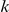 close to 100%, results become statistically insignificant, which is expected, since we choose as GDC-central all genes in the network).

### Dominating sets capture BC genes, signaling pathways, and drug targets

**Figure 8:**
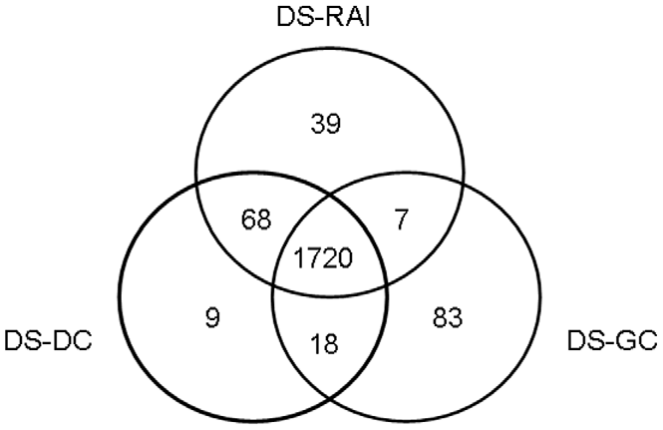
Overlap of the three DSs created by DS-RAI, DS-DC, and DS-GDC algorithms applied to the human PPI network.

We find DSs in the human PPI network by using the three DS algorithms described above, DS-RAI, DS-DC, and DS-GC (see Methods). We find that the overlap between the three resulting DSs is large, containing 1,720 nodes, out of the total of 1,834 nodes in DS-RAI, 1,815 nodes in DS-DC, and 1,828 nodes in DS-GC DSs (Figure 8). Both of our algorithms, DS-DC and DS-GDC, produce smaller DSs than DS-RAI. Also, each of them produces a DS that captures a huge portion of the DS produced by DS-RAI. Using GDC to guide our algorithm does not seem to result in a smaller DS then when we use DC and thus, we continue our analysis on the DS created by DS-DC.

**Figure 9:**
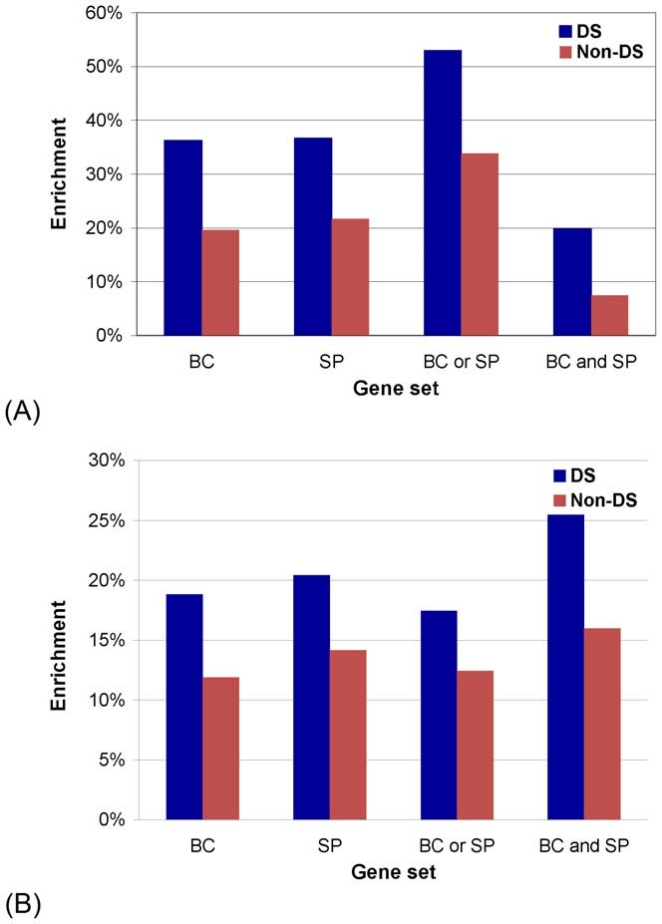
“Biological centrality” of the DS. (**A**) Enrichment in BC and SP genes of the dominating set (“DS”) and its complement (“non-DS”) in the human PPI network. (**B**) Enrichment in drug targets of BC and SP genes that are in the dominating set (“DS”) and BC and SP genes that are not in the dominating set (“Non-DS”).

For the DS created by DS-DC algorithm and for its complement (the set of proteins in the network that are not in the DS, “non-DS”), we calculate their enrichments in BC genes, genes that are members of signaling pathways (SP), genes that are in the union of BC and SP genes (“BC or SP”), and genes that are both BC and SP genes (“BC and SP”). We find that the enrichments are much higher for the DS than for non-DS (Figure 9 A). Furthermore, the enrichments for the DS are statistically significant, with 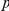-values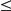 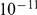, while for non-DS they are not, with 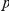-values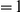 (see Methods).

Furthermore, we measure the enrichment in drug targets of BC and SP genes (i.e., of gene categories “BC”, “SP”, “BC or SP”, and “BC and SP” defined above) that are in the DS, and of BC and SP genes that are not in the DS. If our hypothesis that the topological positioning of nodes in the DS indeed reflects their functional importance is correct, then BC and SP genes that are in the DS should contain more drug targets than BC and SP genes that are not in the DS, since proteins that are targeted by drugs are clearly important for normal cellular functioning. Indeed, we find that enrichments in drug targets are much higher for BC and SP genes that are in the DS than for BC and SP genes that are not in the DS (Figure 9 B). Furthermore, these enrichments for the BC and SP genes that are in the DS are statistically significant, with 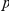-values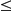 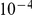, while for SP and BC genes that are not in the DS they are not, with 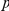-values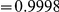 (see Methods).

### Functional analysis of topologically central genes

For each category of BC genes (A, C, HIV, and PI genes), we compute enrichment of GDC-central and non-GDC-central genes in each of the Gene Ontology (GO) terms [71]. We consider all GO terms belonging to each of the three GO categories: molecular function (MF), biological process (BP), and cellular component (CC). Of the total of 1,359 MF, 3,925 BP, and 736 CC GO terms present in the human PPI network, 117 MF, 379 BP, and 27 CC GO terms are statistically significantly enriched (see Methods) in all 4 BC gene categories of GDC-central genes, while 4 MF, 10 BP, and 4 CC GO terms are statistically significantly enriched in non-GDC-central genes. Interestingly, there is no overlap between GO terms that are enriched in central genes and GO terms that are enriched in non-central genes.

Similar results are obtained for central and non-central genes with respect to membership in the dominating set (DS). DS-central genes are statistically significantly enriched in 153 MF, 574 BP, and 44 CC GO terms, while non-DS-central genes are statistically significantly enriched in 7 MF, 7 BP, and 0 CC GO terms, with no overlap between GO terms of central and non-central genes.

Hence, central genes appear to group by functions that are different than functions of non-central genes. Biological functions with the most significant enrichments that are present among all groups of central genes (but none of which is present among any of the groups of non-central genes) include many processes critical for normal cellular functioning, such as: regulation of cell cycle, apoptosis, multicellular organism growth, telomere maintenance, innate immune response, regulation of cell differentiation, signal transduction, activity of many signaling pathway cascades (e.g., MAPK, I-kappaB kinase/NF-kappaB, EGFR, FGFR, IGFR, androgen receptor, nerve growth factor receptor, T cell receptor, toll-like receptor, etc.), phosphorylation, response to DNA damage, blood coagulation, regulation of cell proliferation, T cell activation and co-stimulation, response to tumor necrosis factor, response to drug, interspecies interaction between organisms etc.

### Implications

GDC captures the density and topological complexity of up to 4-deep network neighborhood around a node. Since we have demonstrated significant enrichment of GDC-central proteins in BC genes, this means that genes that are involved in key biological processes occupy topologically complex and dense parts of the human PPI network. Similarly, since we have demonstrated significant enrichment of DSs in BC and SP genes, this indicates that proteins that are vital for normal cellular functioning reside on the “spine” of the network that dominates, i.e., connects, all other parts of the network. Hence, the notion of network domination seems to capture the topology required for passing cellular signals efficiently throughout the network.

We hypothesize that GDC-central proteins and proteins in DSs of PPI networks could represent potential candidates for therapeutic intervention, since targeting GDC-central proteins with drugs would have more significant impacts on the network than targeting proteins that reside in sparse and non-complex network regions and since the topology of a DS can enable quick propagation of drug effects through the entire network. Indeed, we find that the enrichment in drug targets of genes that are GDC-central *or* are in the DS (this is the union of the set of genes that are GDC-central and the set of genes that are in the DS) is 11.4% and it is statistically significant, with 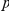-value of 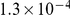. Furthermore, the enrichment in drug targets of genes that are simultaneously GDC-central *and* are in the DS (this is the intersection of the set of genes that are GDC-central and the set of genes that are in the DS) is even higher, it is 31.7%; this enrichment is also statistically significant, with 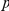-value of 0. Hence, not only that each of the two concepts of topological centrality, GDC and DS, captures a statistically significant percentage of drug targets, but also when the two centralities are combined, the percentage of drug targets that they capture is significant and even higher.

### Concluding remarks

We propose a new centrality measure, graphlet degree centrality (GDC), to simultaneously measure the density and complexity of a node's extended neighborhood by counting the number of different graphlets that the node touches. We find that: (1) the enrichments in BC genes are much higher for GDC-central genes than for non-GDC-central genes; (2) the observed enrichments are statistically significant for GDC-central genes, while for non-GDC-central genes they are not; (3) BC genes that are GDC-central have higher and statistically significant enrichments in known drug targets than BC genes that are non-GDC-central; and (4) GDC outperforms other centrality measures in the sense that it uncovers the largest number of BC genes among the most central genes and is thus the most discriminative centrality measure.

Given the topologically central role of nodes in a DS, we apply to the human PPI network an existing DS algorithm that is commonly used in telecommunications, with the hypothesis that a DS might capture a set of proteins in a PPI network that are involved in important biological processes and mechanisms crucial for cell vitality. Also, we design a new and simpler DS algorithm that outperforms the existing algorithm on our data. We emphasize that our main focus is not to create a state-of-the-art algorithm for finding DSs, but instead, to demonstrate, as a proof of concept, that a DS of a PPI network found by a very simple algorithm captures biologically vital proteins. Indeed, we find that: (1) the enrichments in BC and SP genes are much higher for nodes of DSs than for nodes outside of DSs; (2) the observed enrichments are statistically significant for nodes of DSs, while for nodes outside of DSs they are not; (3) BC and SP genes that are in DSs have much higher and statistically significant enrichments in known drug targets than BC and SP genes that are not in DSs; and (4) GDC-central genes that are also in the DS contain the highest, statistically significant percentage of drug targets.

These results imply that nodes in dense and complex neighborhoods that dominate the network are vital for normal cellular functioning and signaling. Hence, they might be targets for new therapeutic exploitation. Further algorithmic improvements would aid in more precise identification of these new targets.
